# Experimental hut to study the indoor behaviour and effects of insecticide-treated bednets on phlebotomine sand flies (Diptera: Psychodidae)

**DOI:** 10.1590/0074-02760180131

**Published:** 2018-07-19

**Authors:** Olga Lucía Cabrera, Erika Santamaría, Raúl Hernando Pardo

**Affiliations:** Instituto Nacional de Salud, Grupo de Entomología, Bogotá, Colombia

**Keywords:** sand flies, Lutzomyia, experimental huts, lambda-cyhalothrin, deltamethrin, induced exophily

## Abstract

Behavioural effects of insecticides on endophagic phlebotomine sand fly vectors of *Leishmania* are poorly understood mainly because of the lack of an experimental hut (EH) in which to study them.

**OBJECTIVE:**

To build an EH to evaluate the effects of long-lasting insecticide-treated nets (LLINs) on *Lutzomyia longiflocosa*.

**METHODS:**

The study had two phases: (1) Laboratory experiments using tunnel tests to select the traps for the EH; and (2) EH construction and evaluation of the effects of deltamethrin and lambda-cyhalothrin LLINs on *L. longiflocosa* females inside the EH.

**FINDINGS:**

Phase 1: The horizontal-slit trap was the best trap. This trap collected the highest percentage of sand flies, and prevented them from escaping. Therefore, this trap was used in the EH. Phase 2: The main effects of LLINs on *L. longiflocosa* in the EH were: landing inhibition, inhibition from entering the bednet, induced exophily, and high mortality (total and inside exit traps).

**CONCLUSIONS:**

The EH was effective for evaluating the effects of LLINs on endophagic sand flies. Although both types of LLINs showed high efficacy, the lambda-cyhalothrin-treated LLIN performed better. This is the first report of induced exophily in sand flies.

An experimental hut (EH) is a room of standard size and shape that simulates a human dwelling. Such EHs are used to determine the toxic and behavioural effects of insecticides by monitoring and catching the hematophagous Diptera that enter, exit, feed, and die within them.^1^ Compared with real houses, EHs have the advantage of enabling the collection of insects, and are uniform in terms of structure, size, and the number and location of openings, which affect the density of insects. The following effects of insecticides on mosquitoes have been identified using EHs: (a) insecticide-induced mortality, (b) deterrence, (c) induced exophily, and (d) blood-feeding inhibition.^1^

Initially, EHs were used to evaluate the efficacy of indoor residual spraying (IRS) against malaria vector mosquitoes of the genus *Anopheles*. Then, EHs were used to evaluate the effectiveness of insecticide-treated bednets.^2^ EHs have also been used to determine the repellent effect of insecticides and repellents on *Anopheles* and *Aedes* mosquitoes.^3^ ^-^ ^5^ The World Health Organization recommends the use of EHs in phase II (small-scale field trial) of the evaluation process of new insecticides intended for IRS and long-lasting insecticide-treated nets (LLINs).^1^ EHs have also been proposed as important tools for studying the effects of push-pull control strategies involving spatial repellents and lure traps.^6^

Generally, an EH consists of a room with a door, windows, and openings located in the upper (eave openings) and lower parts of the walls. Systems that trap insects when entering or exiting the EH can be attached to the openings. EHs have been used for more than 70 years, during which time several models of huts have been built.^2^ ^,^ ^7^ The first EH was built in Kenya in the early 1940s, and was similar in size and shape to the local inhabited huts.^8^ The EH was windowless, with mud walls, a thatched roof, and eave openings. A hessian ceiling was added to facilitate mosquito collection. Then, window exit traps were fitted to collect exiting mosquitoes.^9^ In the mid-1960s, the veranda-type EH was introduced to recover species of mosquitoes that evolved to use the eaves to exit the hut in response to the repellent effect of insecticides.^10^ More recent designs propose the use of portable EHs that have walls made of removable and easy-to-assemble wood plank panels, which enable removal of the traces of insecticides used in the experiments.^11^

Currently, there is no information about the effects of insecticides on phlebotomine sand flies conducted within EHs, as most studies are of malaria vectors. For instance, EHs were used to determine that pyrethroids (permethrin, alpha-cypermethrin, and lambda-cyhalothrin) used for IRS or treating bednets cause high mortality (> 70%) in *Anopheles* mosquitoes.^12^ ^,^ ^13^ Also, DDT, permethrin, alpha-cypermethrin, and lambda-cyhalothrin had deterrent effects, preventing more than 60% of mosquitoes from entering EHs.^12^ ^-^ ^14^ Alpha-cypermethrin and lambda-cyhalothrin stimulated the exit (induced exophily) of more than 80% of *An*. *funestus*, *An. gambiae*, and *An. arabiensis*.^12^ ^,^ ^13^ EHs were also used to show that insecticides caused high variability in blood-feeding inhibition among mosquito species.

EHs have only been used with phlebotomine sand fly vectors of leishmaniasis in one recent study by Kirstein et al.^15^ However, there is urgently needed to understand and evaluate the effects of insecticides on endophagic sand flies. Two reasons could explain the lack of research on endophagic sand flies using EHs. Firstly, the current EHs used for mosquitoes are unsuitable for the specific behavioural and physical features of sand flies. Secondly, the transmission cycle of leishmaniasis is generally a one-way path (in the case of zoonotic leishmaniasis), where humans are the only hosts and the reservoirs are wild or domestic mammals. This could explain the lack of interest in studying the indoor behaviour, particularly the exophilic behaviour, of sand flies.

Although in principle, the EHs designed to study mosquitoes could be used to study phlebotomine sand flies, the differences in size and behaviour between these Dipterans make this difficult. Most problematic is the small size (1.5 to 4 mm, with an *in vivo* height of 2.2 mm) of phlebotomine sand flies, which are approximately one-third the size of mosquitoes.^16^ Since the current traps in EHs have an entrance aperture width greater than 30 mm, which is at least 14 times the height of a sand fly, they do not prevent the escape of sand flies from the EH. The small size of sand flies also makes recovering them from large traps, such as veranda traps, difficult. Phlebotomine sand flies are known to use refuges or escape through very small spaces, making collecting them from within the EH difficult. To address these issues, this study aimed to construct an EH for endophagic sand flies and to validate its efficacy at determining the effects of two LLINs on *Lutzomyia longiflocosa*, a cutaneous leishmaniasis (CL) vector in the sub-Andean region of Colombia.

MATERIALS AND METHODS

The study consisted of two phases: (1) Laboratory experiments; two experiments were performed to select the type of modified exit trap to be used in the EH. In the first experiment, the efficiency of two types of exit traps in capturing phlebotomine sand flies was evaluated. In the second experiment, the efficiency of the same traps in preventing the escape of sand flies was evaluated. (2) Field experiments; EHs were constructed in the rural settlement of Venecia (Huila department, southwestern of Colombia) considering the following elements: (a) the results of the trap tests, (b) previous knowledge of the entering/exiting behaviour of sand flies from real houses, (c) the specific design and materials used in the construction of rural house in the region where the EHs were built, and (d) some of the structural characteristics of the EHs that were previously built for mosquitoes. The EH was effective validated whilst evaluating the effects of two LLINs on *L. longiflocosa*.

Laboratory experiments: selection of a modified exit trap to be incorporated into the EHs for sand flies

*Tested traps* - Two types of exit traps-louvre traps and horizontal-slit traps - with smaller entrance apertures to capture sand flies, were evaluated. The louvre trap^17^ consisted of a set of wooden blades that were vertically stacked with spaces of 3.2 cm between each blade at an inclination angle of 30º, as measured from the base of the trap. When sand flies passed through the trap, they were collected in a cage. To modify the trap for sand flies, the spacing between the blades was reduced 10 times to 0.3 cm with a total number of 10 spaces (Fig. 1A). The horizontal-slit trap^18^ was a rectangular, trapezoidal oblique prism-shaped box leaning on one of its sides, which converged with the parallel face, creating a funnel that ended in a horizontal aperture that was reduced from the conventional 3 cm^7^ to two test sizes, 0.2 and 0.4 cm (Fig. 1B) for sand flies. A collection cage was attached to this system to trap the sand flies.

*Experiment 1: ability of traps to catch phlebotomine sand flies* - In this experiment, the efficiency of the traps that enabled females to enter the EHs was evaluated. The variables compared included the percentage of females that passed through the trap, attracted by animal bait inside an observation tunnel, and the percentage of blood-fed females. In this experiment, the louvre trap was placed in such a manner that the blades were pointed downward relative to the point where the sand flies were released (Fig. 1A_1_); the horizontal-slit trap was placed in such a manner that the mouth of the funnel faced the point where the sand flies were released (Fig. 1B_1_). The sand flies were unfed *Lutzomyia longipalpis* females aged 2-6 days obtained from a laboratory colony at the National Health Institute, Bogotá. Four treatments were evaluated: (1) louvre trap with spacing between slits of 0.3 cm, (2) horizontal-slit trap with spacing of 0.2 cm, (3) horizontal-slit trap with spacing of 0.4 cm, and (4) control, wooden sheet with a central rectangular window (23 cm × 10 cm). For testing, all traps were attached to a sheet support with the same features as the control.

**Fig. 1:**
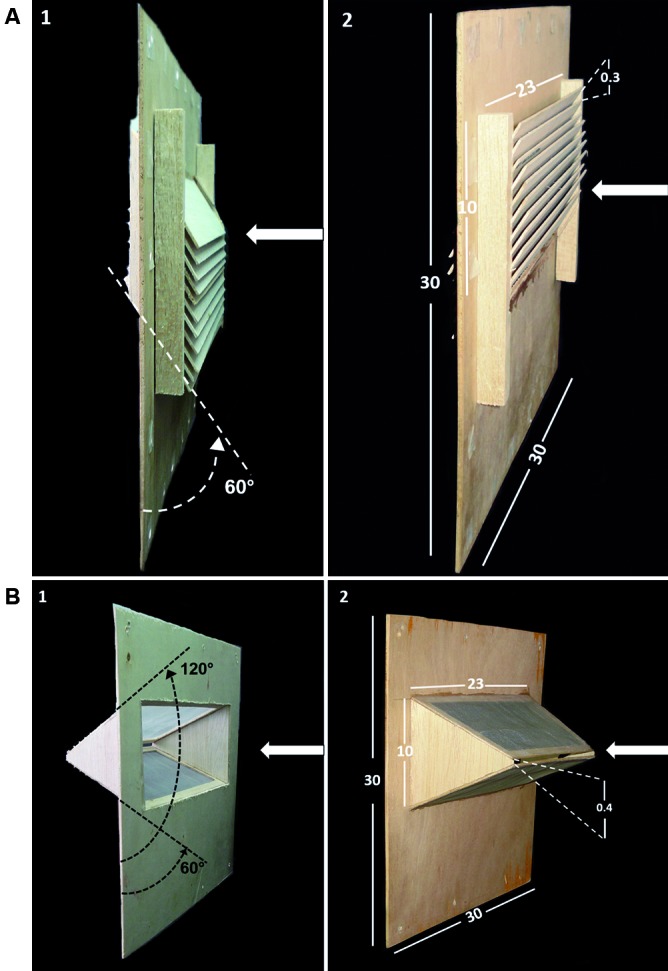
exit traps were attached to wooden sheet supports to test phlebotomine sand flies under laboratory conditions. (A) louvre trap, (B) horizontal-slit trap. 1: position to facilitate entrance of sand flies, 2: position to prevent escape of sand flies. Arrows show the direction where the sand flies come from (i.e., release chamber). Measurements are in centimetres.

The experiment used a randomised design in which treatments were tested successively within a period of 4 h. In each experiment, each of the treatments traps acted as partial barrier inside a transparent acrylic tunnel (length = 60 cm, height = 30 cm, width = 30 cm) to hinder blood-feeding of *L. longipalpis* females. The tunnel consisted of two equally sized sections: a release chamber and a feeding chamber. During the experiment, an anaesthetised golden hamster (*Mesocricetus auratus*) was placed inside the feeding chamber. Twelve *L. longipalpis* males were placed in the chamber to stimulate host seeking by the females. A group of 25 *L. longipalpis* females was placed inside the release chamber. The experiment was conducted in the dark for 90 min, between 11:00 and 15:00 h, and the hamster was replaced after 45 min. When the experiment was concluded, the phlebotomines were removed from the tunnel, and the number of females in each chamber was recorded, along with their blood-feeding condition. The experiment was replicated four times.

*Experiment 2: ability of traps to prevent phlebotomine sand flies from escaping* - This experiment evaluated the traps placed opposite the entrance position, simulating the conditions in which sand flies would retreat to escape from the traps. To achieve this, both traps were rotated 180º around their vertical axes (Fig. 1A_2_ and 1B_2_). The percentage of females that did not pass through the traps was recorded. All other procedures were the same as in experiment 1.

The trap that recorded the greatest percentage of sand flies entering in experiment 1, and the greatest percentage of sand flies not-entering (proxy for non-escaping), in experiment 2, was considered as the most efficient. This trap was used as the exit trap in the EHs.

**Fig. 2:**
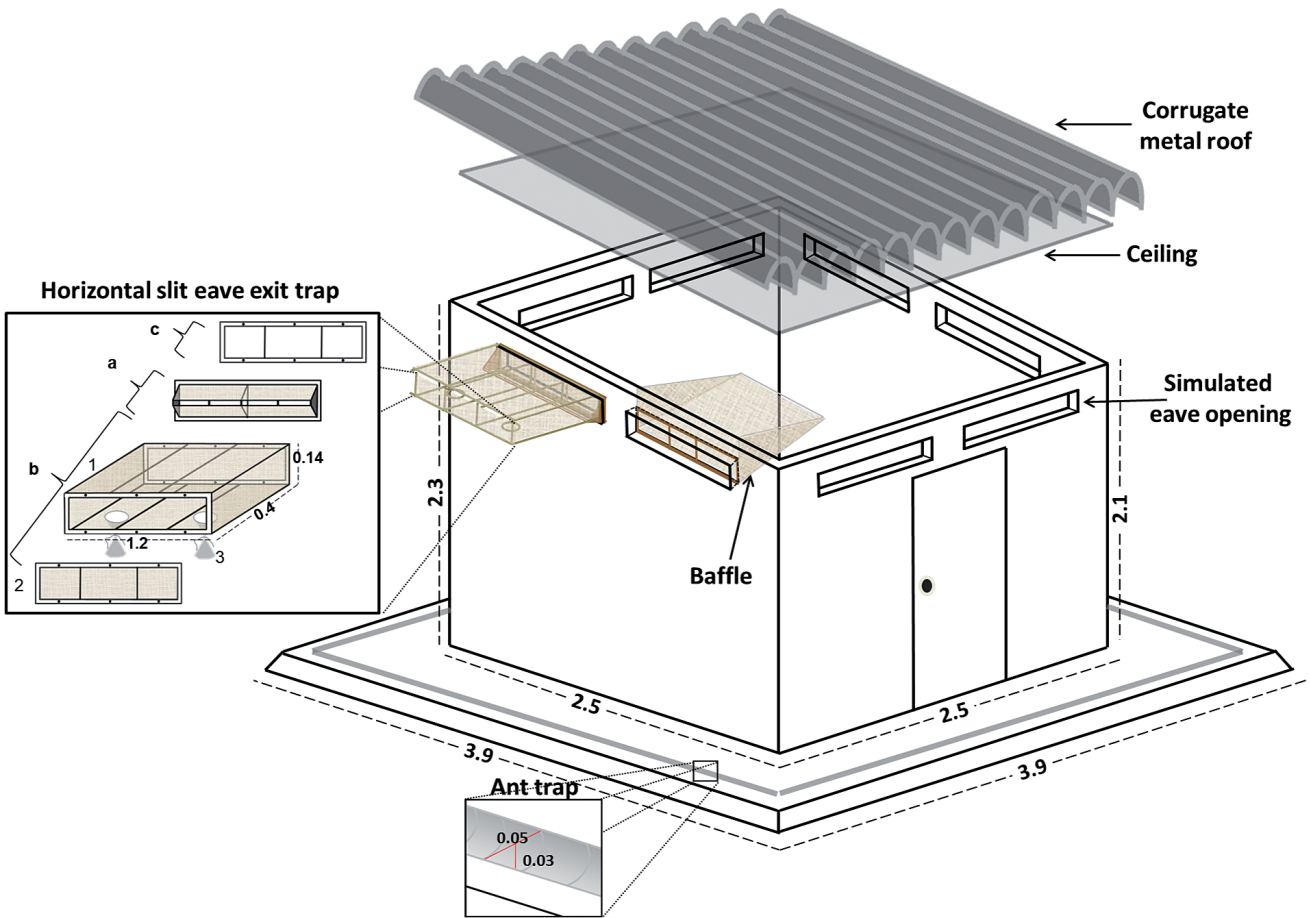
schematic diagram of the experimental hut used to evaluate the effects of insecticides on sand fly behaviour. The roof and ceiling were removed from their usual positions and only traps from one side of the hut are shown to facilitate the view of the simulated eave openings and traps. Horizontal-slit eave exit trap: (A) horizontal-slit trap system, (B) collection cage (1: cage body, 2: cage lid, 3: sleeves), (C) coupling frame. Drawing not to scale, measurements in metres.

Field experiments: hut construction and validation

*Hut construction* - Three EHs were built in the sub-Andean region of Colombia in the dispersed rural settlement of Venecia (2º39′47″ N and 75º14′31″ W, map datum WGS 84), Campoalegre County, Department of Huila. Each EH was built next to a house with historically high abundance of the sand fly *L. longiflocosa*, the main vector of CL in the region. The EHs were located 500 to 1,500 m apart, at approximately 1,600 m a.s.l.

Each EH consisted of a room (2.5 × 2.5 × 2.3 m) with construction design and materials similar to those used in the local houses of the study area^16^: the walls were constructed of brick, and covered with light cloth; the flooring was cement; and the roof was galvanized sheet metal. In the upper part of each of the four walls, two rectangular apertures of 1.15 m × 0.13 m simulated the eave openings (Fig. 2) that were used for ventilation in local houses and as entrance/exit points by the sand flies.^19^ The EH was set up with a baffles system placed on the simulated eave openings to allow the entrance, but not exit, of sand flies^7^ and modified horizontal-slit eave exit traps^18^ to catch the sand flies before they left the EH. These traps (slit width = 0.4 cm) were the most efficient in the laboratory experiments, permitting more than 80% of sand flies to enter and preventing approximately 90% from escaping (passing in the opposite direction to the entering position). Since *L. longiflocosa* enters and exits houses in a non-random pattern^19^, a baffle and a horizontal-slit eave exit trap were placed on the apertures of each wall (Figs 2-3). To prevent sand flies from escaping and to collect them, a drywall ceiling was installed, and the indoor walls and ceiling were painted white. Additionally, every visible aperture or small crack in the EH walls and ceilings was blocked so that sand flies could not escape or hide, as they can pass through very small spaces. To confirm this, groups of females were released inside the EHs and recaptured several hours later, with the eave horizontal-slit exit traps as the only exit. The recapture percentage was between 65% and 69%, which was considered acceptable (unpublished observations by the authors). The hut was surrounded by water channels to prevent loss of sand flies caused by scavenger ants, which has been observed inside the local houses of the study area^16^ (Fig. 2).

*Validation of the experimental hut: toxic and behavioural effects of LLINs on sand flies* - This experiment was performed in each of the three EHs between February and March 2014. The aim was to evaluate the behavioural and toxic effects of two types of LLINs, treated with deltamethrin and lambda-cyhalothrin, on *L. longiflocosa*, a vector of CL in the sub-Andean region of Colombia. Three treatments were evaluated: (1) bednet industrially coated with deltamethrin, 55 mg/m^2^ (Permanet 2.0, Lausanne, Vaud, Switzerland); (2) bednet manually treated (do it yourself) with a slow-release capsule suspension formulation of lambda-cyhalothrin, 60 mg/m^2^, combined with a polymer-binding agent (Icon Maxx^®^, Basel, Basel-Stadt, Switzerland); and (3) untreated bednet. The three bednets were made of polyester, with a mesh size of 2.0 mm and 24 perforations/cm^2^. In each bednet, six 4 × 4 cm holes were made to simulate the conditions of a torn net, two holes on each long side and one on each short side.^1^ To prevent cross contamination with insecticides between huts, the nets were handled with exclusive gloves for each treatment, the floor was covered with paper sheets that were replaced daily, and the EHs were aired out for 6 h during the day.

Before the experiment, each EH was prepared as follows: (1) the canals that surrounded the house were filled with water, (2) the floor was covered using new white paper, which prevented contamination with insecticide and allowed observation of fallen phlebotomine sand flies, (3) the sand flies inside the EH and the exit traps were collected to prevent them from interfering with the sand flies collected during the experiment; and (4) cracks were sealed using masking tape, mainly in the door frame, to prevent sand flies from escaping through other routes to the exit traps. Four horizontal-slit exit traps, one for each wall, were positioned alternately in the eave openings. Entering baffles were installed in the remaining four eave openings.

The study used a 3 × 3 Latin square design (repeated 3 times, n = 9). The three treatments were simultaneously evaluated in each replicate, and each treatment was rotated between all three EHs in each replicate. During the experiment, two adult volunteers remained inside the bednet, and exposed their forearms and legs from their knees to their ankles. The same volunteers remained in the same EH during the study. While one of the volunteers remained still, the other one collected the sand flies as soon as they landed on the exposed parts before they could bite. The experiment lasted for 6 h (20:00-02:00); at the end, the volunteers collected the sand flies and recorded their collection site (inside or outside the bednet or inside the exit traps) and their survival state. The live sand flies were provided with a 30% sugar solution and kept for 24 h, after which delayed mortality was determined. The sand flies were identified in the laboratory using taxonomic keys.^20^

The entomological effects on *L. longiflocosa* females recorded were: (i) human landing inhibition, represented by the reduction in the mean human landing rates inside the insecticide-treated bednets, relative to the control; (ii) inhibition from entering the bednets, evaluated by the reduction in the percentage of females that passed through the insecticide-treated bednets, relative to the control; (iii) induced exophily, evaluated by the increase in the percentage of females found in exit traps and the decrease in the percentage of resting females outside the insecticide-treated bednets, relative to the control; (iv) exit side preference, evaluated by the variation in the percentage of females caught by the exit traps at the front, rear, right, and left sides of the insecticide-treated bednets, relative to the control; (v) deterrent effect, represented by the reduction in the mean number of females entering the insecticide-treated bednets, relative to the control; and (vi) mortality rates immediately and at 24 h after the experiment, including the comparison of 24 h mortality between exiting females caught in the horizontal-slit exit trap and females inside the hut (excluding the exit traps).

*Statistical analysis* - In experiment 1, the percentages of females that passed through the traps were compared using generalised linear models analysis, assuming a bino­mial distribution of errors. Homogeneity of variance and normality of errors was assessed using standardised residuals vs fitted values and quantiles of residuals vs quantiles of normal distribution plots, respectively, and predicted values vs observed data plots. In experiment 2, the percentage of females that did not pass through the traps was initially analysed using the Likelihood ratio Chi-squared test (*L*c^*2*^ ), followed by pairwise comparisons for appropriately collapsed tables. The generalised linear models analysis was not possible for this data due to overdispersion in the binomial model.

The data for the total density and landing rates of females inside the EHs are presented as geometric means (antilogarithms of the mean number of females, previous transformation to natural logarithm, ln ‘x’ ) with their 95% confidence intervals (95% CI). Since none of the response variables transformed well to any of the known distributions (Poisson or normal), they were compared using non-parametric tests. The Kruskall-Wallis test was applied to compare the number of females for more than two treatments, and between two treatments. The Mann-Whitney test was used for unpaired data. The numbers of females inside the nets and exit traps and mortality are presented as percentages with their respective 95% CI and were analysed using Chi-squared (c^2^) tests. Additionally, to evaluate the effects of LLINs on the exiting preferences of females, the percentage of exiting females on each side of the EHs was analysed using a (c^2^) test for one variable. In this test, the observed number of females exiting on specific exit sides (front, rear, left, and right) of the EH was compared with the expected (equal numbers for each side), assuming a random exiting preference. Statistical analyses were performed using Stata 12. The results were considered significant at p < 0.05.

**Fig. 3:**
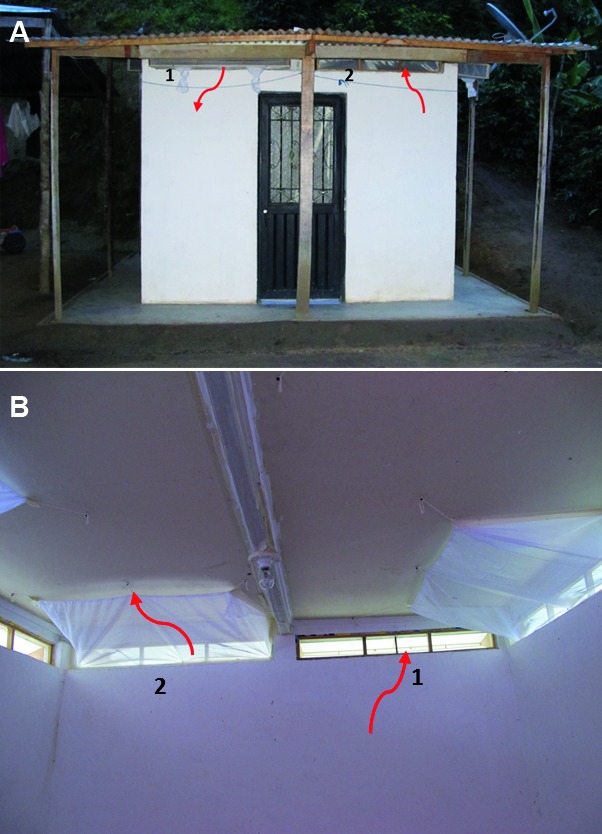
image of the experimental hut. (A) external view showing the front of the horizontal-slit eave exit trap (1) and entering baffle (2), and (B) traps viewed from the inside (arrows show entry and exit movements of sand flies).

**TABLE I:** Evaluation of pass of *Lutzomyia longipalpis* females through traps in the entering position and in the opposite direction within an observational tunnel

Variable	Treatment				Statistic
	Control	Slit 0.4 cm width	Slit 0.2 cm width	Louvre 0.3 cm spacing between blades and 10 spacings	
Trap in the entering position					
Number of released females	102	102	99	98	
Number of females passed through	78	83	46	67	
Percentage of pass through females (CI)	76.5^*a,c*^ (67.0 - 84.3)	81.4^*c*^ (72.4 - 88.4)	46.5^*b*^ (36.4 - 56.8)	68.4^*a*^ (58.2 - 77.4)	F _(3, 8)_ = 29.11, p < 0.001
Number of blood-fed females	67	58	29	45	
Percentage of blood-fed females (CI)	85.9^*a*^ (76.2 - 92.7)	69.9^*a*^ (58.8 - 79.5)	63.0^*a*^ (47.5 - 76.8)	67.2^*a*^ (54.6 - 78.1)	F _(3, 12)_ = 1.90, p = 0.183
Trap in the opposite direction to the entering position					
Number of released females	100	102	101	93	
Number of females did not passed through	23	89	99	48	
Percentage did not pass through females (CI)	23.0 (15.2 - 32.5)	87.3 (79.2 - 93.0)	98.0 (93.0 - 99.8)	51.6 (41.0 - 62.1)	*L*c^2^_(3)_ = 176.60, p < 0.001

CI: 95% confidence interval; *L*c^2^: Likelihood ratio chi-square test. In the F test, each row values with different superscript letters are significantly different (p < 0.05).

*Ethics* - To mitigate the risk of potentially infective sand fly bites, the volunteers wore protective clothing to cover areas of the body not used to collect sand flies and sand flies were collected by highly trained volunteers.^21^ Also, all volunteers signed an informed consent form. The use of the golden hamster as a blood source for the released sand flies was approved by the Ethics Committee of the National Institute of Health of Colombia (Agreement No. 5 Jun 25, 2009).

RESULTS

Laboratory experiments

*Experiment 1: ability of traps to catch phlebotomine sand flies* - The horizontal-slit trap with an aperture of 0.4 cm permitted the greatest number of *L. longipalpis* females to enter, which was 81.4% (83/102), followed by the control, 76.5% (78/102), the louvre, 68.4% (67/98), and the horizontal-slit with an aperture of 0.2 cm, 46.5% (46/99). The difference between treatments was statistically significant (Table I). The percentage of females entering the horizontal-slit trap with an aperture of 0.4 cm, was significantly greater than that entering for the louvre, (z = 2.29, p = 0.022), and the horizontal-slit trap with an aperture of 0.2 cm, (z = 4.63, p < 0.001). However, there was no significant difference in the percentage of sand flies entering through the horizontal-slit trap with an aperture of 0.4 cm and the control (z = 0.37, p = 0.710). Also, although there were apparent differences in the percentage of blood-fed females between treatments, they were not statistically significant (Table I).

*Experiment 2: ability of traps to prevent phlebotomine sand flies from escaping* - When the traps were positioned opposite to the entrance, 98.0% (99/101) of *L. longipalpis* females were prevented from exiting through the horizontal-slit trap with an aperture of 0.2 cm, followed by the horizontal-slit trap with an aperture of 0.4 cm, 87.3% (89/102), the louvre, 51.6% (48/93), and the control, 23% (23/100). The difference between treatments was statistically significant (Table I). The percentage of females that did not pass through all combined traps was significantly greater than the control (*L*c^*2*^ = 104.47, p < 0.001). The percentage of females that did not pass through the horizontal-slit traps with apertures of 0.2 cm plus 0.4 cm was significantly greater than the percentage that did not pass through the louvre trap (*L*c^*2*^ = 62.59, p < 0.001). Finally, the percentage of females that did not pass through the horizontal slit trap 0.2 cm was significantly greater than the percentage of females that did not pass through the horizontal slit trap 0.4 cm (*L*c^*2*^ = 9.54, p = 0.002).

Field experiments

*Behavioural effects of the LLINs on phlebotomine sand flies* - Over nine sampling nights, a total of 2,102 insects of the *Lutzomyia* genus entered the three EHs. All were *L. longiflocosa*, except one female that belonged to the subgenus *Helcocyrtomyia*. Since *L. longiflocosa* females represented 98.7% (2,073/2,101) of the specimens captured, only data for this sex were analysed. The effects of the LLINs on the behaviour of *L. longiflocosa* are shown in Tables II-III.

*Human landing inhibition* - The LLINs caused a significant reduction in the landing rates of *L. longiflocosa* on human bait. The landing rate in the lambda-cyhalothrin-treated LLIN was one tenth that of the control, 4.9 vs 49.5 females/night/2 persons, respectively (z = 3.58, p = 0.0009), which corresponded to a 90.1% reduction in landing rate. For the deltamethrin-treated LLINs, the rate was one third that of the control, 15.1 vs 49.5, respectively, with a marginally significant difference (z = -2.34, p = 0.058) and a reduction percentage of 69.5%. There was no significant difference in the landing rate between the two types of LLINs (z = -2.21, p = 0.081).

*Inhibition from entering the bednet* - The LLINs reduced the number of *L. longiflocosa* entering the bednets. The percentages of females that passed through the lambda-cyhalothrin-treated, 13.9%, and deltamethrin-treated, 44.8%, LLINs were significantly lower than the control, 77.1% (c^*2*^ = 492.2, p < 0.001 and c^*2*^ = 179.60, p < 0.001, respectively), which was equivalent to a reduction of 82.0% and 41.9%, respectively. The difference in the percentages of sand flies entering the two types of treated bednets was highly significant (c^*2*^ = 114.9, p <0.001).

*Induced exophily* - The percentage of females found in the modified horizontal-slit exit traps in the lambda-cyhalothrin, 35.8%, and deltamethrin, 19.5%, treatments were significantly greater than that in the control, 6.3% (c^*2*^ = 199.55, p < 0.001 and c^*2*^ = 66.13, p <0.001, respectively). The difference between the two types of LLINs was also significant (c^*2*^ = 36.89, p <0.001). Conversely, the percentage of females resting outside the LLINs (in relation to the total number of females outside the bednets) was significantly reduced in the lambda-cyhalothrin-treated, 23.3%, and deltamethrin-treated, 30.5%, LLINs compared with the control, 72.3% (c^*2*^ = 137.1, p < 0.001 and c^*2*^ = 97.2, p < 0.001, respectively). This corresponded to a reduction in resting females of 67.8% and 57.8%, respectively. The difference in the percentages of resting females between the two types of LLINs was also statistically significant (c^*2*^ = 4.82, p = 0.028).

*Exit sides preferences* - Regardless of the treatments, there was a highly significant difference in the percentage of *L. longiflocosa* females (sum of all treatments) exiting different sides of the EHs (c^2^ = 106.41, *df* = 3, p < 0.001). The greatest percentage (46.7%) of females exited from the front of the EH, followed by the right side (27.2%), while smaller percentages of females exited by the rear (12.9%) and left sides (13.2%) (Table III). However, there was no significant difference in the percentages of females exiting from different sides of the EHs between treatments (c^2^= 8.54, *df* = 6, p = 0.201). However, there was a highly significant difference in the total percentage of females (sum of all treatments) exiting the EHs by the sides (c^*2*^ = 106.41, gl = 3, p < 0.001), with the highest percentage of females exiting from the front side, 46.7% (95% CI: 41.4-52.1) (Table III).

*Deterrent effect* - It seemed the LLINs dissuaded *L. longiflocosa* females from entering the EHs, as there was a reduction in the total density of females (Table II), from 74.2 females/night in the EHs with the control treatment to 52.6 females/night in the EH with deltamethrin-treated LLIN and 43.1 females/night in the EH with lambda-cyhalothrin-treated LLIN. However, the difference between treatments (including the control) was not significant (c^*2*^ = 0.998, *df* = 2, p = 0.607).

Toxic effects of the LLINs on phlebotomine sand flies

*Mortality immediately after the experiment* - The LLINs treated with lambda-cyhalothrin caused a total immediate mortality in *L. longiflocosa* females of 52.5% (95% CI: 47.7-57.3), almost twice that of the LLINs treated with deltamethrin, 28.6% (95% CI: 25.2-32.2), which was significantly different (c^*2*^ = 64.49, p < 0.001). In the control, there was almost no mortality immediately after the experiment, 0.1% (95% CI: 0-0.6).

*Mortality 24 h post-experiment* - The mortality of *L. longiflocosa* at 24 h post-exposure was very high, 82.6% (95% CI: 79.5-85.4), for the LLIN treated with deltamethrin and 78.8% (74.6-85.2) for the LLIN treated with lambda-cyhalothrin, although they were not significantly different from each other (c^*2*^ = 2.53, p = 0.112). In the control, the 24 h mortality was just 4.7% (3.4-6.2).

**TABLE II:** Main behavioral effects of insecticide treated bednets on *Lutzomyia longiflocosa* females (total number = 2,073) in experimental huts (n = 9)

Effect	Variable	Control	Deltamethrin 55 mg/m^2^	Lambda-cyhalothrin 60 mg/m^2^	Chi-square test or (Kruskal Wallis)
Human landing inhibition					
	Total number of females in human landing catches	727	288	55	
	GM number of females/night/2 persons	49.5^*a*^ (24.3 - 99.9)	15.1^*a,b*^ (5.6 - 38.1)	4.9^*b*^ (2.3 - 9.6)	c^2^ = 15.49, *df* = 2, p = 0.0004
	Percentage reduction in landing females		69.5	90.1	
Inhibition from entering the bednet					
	Total number of females inside the bednet	743	301	61	
	Percentage of females inside the bednet (CI)	77.1^*a*^ (74.4 - 79.8)	44.8^*b*^ (41.0 - 48.6)	13.9^*c*^ (10.8 - 17.5)	c^2^ = 512.5, *df* = 2, p < 0.001
	Percentage reduction in females inside the bednets		41.9	82.0	
Exophily					
	Total number of females in exit traps	61	131	157	
	Percentage of exiting females (CI)	6.3^*a*^ (4.9 - 8.0)	19.5^*b*^ (16.6 - 22.7)	35.8^*c*^ (31.3 - 40.5)	c^2^ = 192.28, *df* = 2, p < 0.001
	Number of times of percentage increase in exiting females		3.1	5.7	
	Percentage of females resting outside the bednet (CI)	72.3^*a*^ (65.8 - 78.1)	30.5^*b*^ (25.8 - 35.4)	23.3^*c*^ (19.1 - 27.8)	c^2^ = 154.1, *df* = 2, p < 0.001
	Percentage reduction in resting females		57.8	67.8	
Deterrence					
	Total number of caught females	963	672	438	
	GM number of females/night (CI)	74.2^*a*^ (39.4 - 139.0)	52.6^*a*^ (27.5 - 99.7)	43.1^*a*^ (27.4 - 67.6)	c^2^ = 0.998, *df* = 2, p = 0.607
	Percentage reduction in females		29.1	41.9	

GM: geometric mean; (CI): 95% confidence interval. In each row, values with different superscript letters are significantly different (p < 0.05).

**TABLE III:** Effect of insecticide treated bednets on the exiting preferences of *Lutzomyia longiflocosa* females

Bednet treatment	Side of the hut					Total
	Front				Rear				Left				Right			
	No. females	Percentage (CI)			No females	Percentage (CI)			No. females	Percentage (CI)			No. females	Percentage (CI)		
Control	26	42.6	(30.0 - 55.9)		11	18.0	(9.4 - 30.0)		9	14.8	(7.0 - 26.2)		15	24.6	(14.5 - 37.3)	61
Deltamethrine 55 mg/m^2^	56	42.7	(34.1 - 51.7)		12	9.2	(4.8 - 15.5)		22	16.8	(10.8 - 24.3)		41	31.3	(23.5 - 40.0)	131
Lambda-cyhalothrine 60 mg/m^2^	81	51.6	(43.5 - 59.6)		22	14.0	(9.0 - 20.4)		15	9.6	(5.4 - 15.3)		39	24.8	(18.3 - 32.4)	157
Total	163	46.7	(41.4 - 52.1)		45	12.9	(9.6 - 16.9)		46	13.2	(9.8 - 17.2)		95	27.2	(22.6 - 32.2)	349

CI: 95% confidence interval.

*Mortality of exiting females caught in the exit trap vs females inside the hut* - For the LLINs treated with lambda-cyhalothrin, there was no difference in the 24 h mortality between females caught in the exit traps, 76.4%, and the females inside the hut (excluding the exit traps), 80.1% (c^*2*^ = 0.80, *df* = 1, N = 438, p = 0.372). For the LLINs treated with deltamethrin, the 24 h mortality of females caught in the exit traps was significantly lower, 67.9%, than that of females inside the EHs, 86.1% (c^*2*^ = 24.29, *df* = 1, N = 672, p < 0.001), which corresponded to a 21.1% reduction in mortality. In the control, the 24 h mortality in the exit traps and inside the EHs was minimal, 1.6% and 4.9%, respectively, and they were not significantly different from each other (Fisher exact test = 0.355) (Fig. 4).

DISCUSSION

In this study, an EH for sand flies was built and validated by determining the effects of two LLINs on the sand fly *L. longiflocosa*. The success of the EH was primarily due to several fundamental aspects that affected its operation. The most significant aspect was the selection and adaptation of the most efficient exit trap (laboratory experiments in this study) and knowledge of the entering and exiting behaviour of sand flies. Therefore, the implementation of EHs in other regions should consider possible adjustments related to the behaviour of the target species.

Validation of the experimental hut

*Behavioural effects of LLINs on sand flies* - *Human landing inhibition* - Both types of LLINs affected the behaviour of sand flies. The most important effect was protection against bites from *L. longiflocosa*. The reduction in human landing rate was 90.1% and 69.5% in LLINs treated with lambda-cyhalothrin and deltamethrin, respectively. This protection may be even higher for intact bednets (i.e., without holes). This result concurs with the findings of a few previous studies, where lambda-cyhalothrin-treated bednets caused a greater reduction in human landing rate than deltamethrin-treated bednets. In Colombia, lambda-cyhalothrin (25 mg/m^2^) treated bednets reduced the human landing rate of *L. longiflocosa* by 94%.^22^ In Sudan, the human landing rate of *P. orientalis* decreased from 6.9 females/person/night for an untreated bednet to zero for a lambda-cyhalothrin (10 mg/m^2^)-treated bednet.^23^ A study in southwestern Colombia found that the human landing rate of *L. youngi* inside a bednet treated with deltamethrin (26 mg/m^2^), decreased by 62%, compared with the control.^24^ These studies used lower concentrations of pyrethroids, but intact bednets. The reduced landing rate of *L. longiflocosa* may partly be explained by the toxic effect of the LLINs, as the immediate mortality of *L. longiflocosa* in the EHs was 52.5% and 28.6% in EHs with lambda-cyhalothrin and deltamethrin, respectively, while it was negligible in the control treatment, 0.1%.

**Fig. 4:**
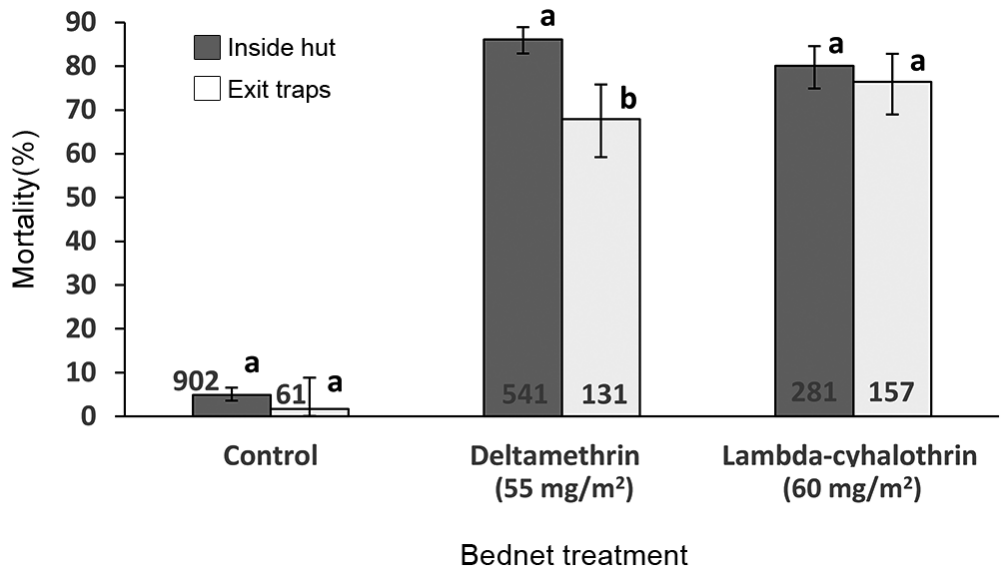
effect of insecticide-treated bednets on 24 h post-exposure mortality of *Lutzomyia longiflocosa* females calculated from the site of collection in the experimental hut. Total number of females is shown in the base of each bar. Error bars show 95% confidence intervals. In each treatment, bars with different letters are significantly different (p < 0.05).

*Inhibition of passage through the bednet* - The significant inhibition of passage of *L. longiflocosa* through the pyrethroid-treated bednets contrasts with the results of a previous study, in which no significant differences were found in the percentages of *L. longiflocosa* females inside a lambda-cyhalothrin-treated bednet and the control.^22^ However, the smaller mesh size in that study could make the measurement of this effect difficult, as only 8.5% of the females passed through in the control.^22^ The reduced passage of *L. longiflocosa* might be attributed to the irritant, in addition to toxic, effect of lambda-cyhalothrin and deltamethrin. Bioassays using World Health Organization cones showed that both pyrethroids caused a significantly shorter contact time of *L. longiflocosa* females with treated bednets compared with the control.^25^ The greater inhibition of passage of *L. longiflocosa* caused by the lambda-cyhalothrin-treated bednet compared with the deltamethrin-treated bednet might be due its greater irritant effect on this sand fly species.^25^

*Induced exophily* - Induced exophily in phlebotomine sand flies caused by insecticides has not been previously reported. This is because exophily is only measured in EHs, which have only been previously used once with sand flies, without exit traps.^15^ The apparent strong induced exophily caused by the lambda-cyhalothrin and deltamethrin LLINs should be taken with caution, as this sand fly is an exophilic species that exits houses after midnight.^19^ Therefore, induced exophily is inherently difficult to observe in this species. Considering that the experimental period (20:00-02:00) excluded dawn, it is probable that the observed induced exophily was the result of an early exit from the EHs by the sand flies. In mosquitoes, several studies have measured exophily caused by pyrethroid-treated bednets. On the Ivory Coast, LLINs treated with lambda-cyhalothrin (Icon Maxx^®^) induced exophily in 73.8% of *A. gambiae* that entered the EHs^26^, whereas LLINs treated with deltamethrin (Permanet^®^) induced exophily in 48% of *C. quinquefasciatus* females.^27^ Both exit percentages were significantly greater than the control. For *L. longiflocosa*, further evidence of exophily caused by LLINs was the significantly reduced percentage of females resting outside the treated bednets compared with the control. This induced exophily could have been due to an irritant and/or repellent effect of the active ingredients of the LLINs on *L. longiflocosa*. The irritant effect (movement of the insects away from a treated surface after making tarsal contact)^28^ was evidenced by the high mortality (67.9-76.4%) of females in the exit traps 24 h post-exposure. These females must have touched the LLINs and tried to move away, becoming trapped in the exit traps. Although there are no known studies of the repellent effect (movement of insects away from a treated surface without making tarsal contact)^28^, for sand flies, this effect cannot be ruled out. The low percentage of live females in the exit traps 24 h post-exposure to the LLINs might have represented a repellent effect. Repellent effects of pyrethroid and other insecticides have been observed in mosquitoes.^29^

*Exit side preferences* - The lack of significant preferences for exit sides of the EHs across treatments indicated that the active ingredients of the LLINs (lambda-cyhalothrin and deltamethrin) did not affect the exit orientation of *L. longiflocosa* females. To our knowledge, there are no previous studies of this effect either in sand flies or mosquitoes. However, our results indicated that *L. longiflocosa* exited the EHs in a non-random pattern, preferring the front side. This result concurs with the findings of a previous study on the entry and exit behaviour of *L. longiflocosa* in rural dwellings at the same study site, in which the number of females exiting and entering the front of the house was significantly higher than at the rear.^19^ This preference for the front side might be explained by the location of the EHs in relation to the mountain and the orientation of the bait odour from the EHs. Furthermore, EHs were positioned so that the front side faced downhill, while the rear side faced upslope, similar to most houses in the study area. Considering that katabatic winds flow downslope at night-time in the Andean region, it is possible that the odour plume would form mainly on the downwind side of the house. Hence, a greater number of females would be expected to exit through the front side (downwind side) of the EHs. We could not test this hypothesis as the predominant wind direction during night-time was not recorded in this study. The effects of other environmental variables (e.g., location in relation to nearest forests, number of domestic animals and persons in the nearest house) on exit preferences appeared to be negligible. Female sand flies probably exited the EHs orientated primarily by the bait odour.

*Deterrent effect* - Although there was no significant reduction in the density of *L. longiflocosa* females entering the EHs with LLINs, a deterrent effect cannot be ruled out. In a previous study in the same area, it was shown that a LLIN treated with lambda-cyhalothrin significantly reduced the density (18.8 females/person/3 h to 7.9 females/person/3 h) of *L. longiflocosa* in bedrooms; however, the effect was attributed to limitations of the sampling method.^22^ There is no report of the deterrent effect of deltamethrin against sand flies. In a similar study in Valle del Cauca, Colombia, no significant difference was found in the density of *L. youngi* between bedrooms with a bednet treated with deltamethrin and an untreated bednet.^24^ In the Judean Desert, no differences were found between the pre-treatment vs. post-treatment density of sand flies (mainly *Phlebotomus sergenti*) in an EH in which the internal walls were lined with a deltamethrin-treated fabric (4.4 g/kg).^15^ In a study at the Ivory Coast, LLINs in an EH that were treated with lambda-cyhalothrin also produced a strong dissuasive effect on *Anopheles* spp., as 76% of the total number of captured mosquitoes were from the control EH.^26^

*Toxic effects of LLINs on sand flies* - The mortality at 24 h post-experiment of LLINs treated with deltamethrin and lambda-cyhalothrin on *L. longiflocosa* was 82.6% and 78.8%, respectively, indicating a high efficacy of these control measures. A lower mortality effect was observed for bednets treated with deltamethrin (25%) against *L. longipalpis* in Brazil, where the 24 h mortality of sand flies outside the treated bednet was 67.7%, compared with 0.4% outside the control.^30^ Bednets treated with lambda-cyhalothrin (25 mg/m^2^) showed a higher 24 h mortality of 99.2% in *L. longiflocosa*. However, this could have been an overestimate, as there was also high mortality (48%) in the control.^22^ The high mortality (24 h post-exposure) of *L. longiflocosa* caused by the two LLINs under semi-controlled conditions in the EHs in this study indicated high efficacy of this control measure.

The mortality (24 h post-exposure) of females collected in the exit traps was high in general, 76.4% and 67.9% for bednets treated with lambda-cyhalothrin and deltamethrin, respectively. This showed that most females had contact with the LLINs long enough to receive a lethal dose of insecticide before exiting the EH. The high mortality of exiting females could reduce the biting rates in nearby houses. This is the first study to report mortality of female sand flies attempting to exit the EHs after exposure to insecticides.

Finally, this study showed the utility of EHs for evaluating the effects of LLINs on sand flies that transmit leishmaniasis. Both types of LLINs (lambda-cyhalothrin and deltamethrin) showed high efficacy against *L. longiflocosa*, causing important toxic (high mortality) and behavioural (reduced landing rates and induced exophily) effects. Lambda-cyhalothrin showed a higher efficacy than deltamethrin, as it caused significantly higher immediate mortality, and increased exophily, whilst reducing the percentages of females entering the bednet, and resting outside the bednets. Also, mortality in the exit traps of the lambda-cyhalothrin-treated LLINs was not significantly reduced compared with that in the EHs. These results can help to predict the effects of large-scale-LLIN interventions for the control of *L. longiflocosa* and other phlebotomine sand flies with similar ecological and behavioural features.

## References

[1] World Health Organization (2006). Guidelines for testing mosquito adulticides for indoor residual spraying and treatment of mosquito nets.

[2] Service MW, Service MW (1993). Experimental hut techniques for evaluating insecticides. Mosquito ecology: field sampling methods. 2nd.

[3] Manda H, Shah P, Polsomboon S, Chareonviriyaphap T, Castro-Llanos F, Morrison A et al (2013). Contact irritant responses of Aedes aegypti using sublethal concentration and focal application of pyrethroid chemicals. PLoS Negl Trop Dis.

[4] N&apos;Guessan R, Rowland M, Moumouni TL, Kesse NB, Carnevale P (2006). Evaluation of synthetic repellents on mosquito nets in experimental huts against insecticide resistant Anopheles gambiae and Culex quinquefasciatus mosquitoes. Trans R Soc Trop Med Hyg.

[5] Tambwe MM, Mbeyela EM, Massinda BM, Moore SJ, Ferreira M (2014). Experimental hut evaluation of linalool spatial repellent agar gel against Anopheles gambiae sensu stricto mosquitoes in a semi-field system in Bagamoyo, Tanzania. Parasit Vectors.

[6] Wagman JM, Achee NL, Grieco JP (2015). Insensitivity to the spatial repellent action of transfluthrin in Aedes aegypti a heritable trait associated with decreased insecticide susceptibility. PLoS Negl Trop Dis.

[7] Okumu FO, Moore J, Mbeyela E, Sherlock M, Sangusangu R, Ligamba G (2012). A modified experimental hut design for studying responses of disease-transmitting mosquitoes to indoor interventions the ifakara experimental huts. PLoS One.

[8] Haddow AJ (1942). The mosquito fauna and climate of native huts at Kisumu, Kenya. Bull Entomol Res.

[9] Muirhead-Thomson RC (1947). The effects of house spraying with pyrethrum and with DDT on Anopheles gambiae and A melas in West Africa. Bull Entomol Res.

[10] Smith A (1965). A Verandah-Trap Hut for studying the house-frequenting habits of mosquitos and for assessing insecticides II the effect of dichlorvos (DDVP) on egress and mortality of Anopheles gambiae giles and Mansonia uniformis (Theo) entering naturally. Bull Entomol Res.

[11] Achee NL, Grieco JP, Andre RG, Rejmankova E, Roberts DR (2005). A mark-release recapture study using a novel portable hut design to define the flight behavior of Anopheles darlingi in Belize, Central America. J Am Mosq Control Assoc.

[12] Malima RC, Magesa SM, Tungu PK, Mwingira V, Magogo FS, Sudi W (2008). An experimental hut evaluation of olyset nets against anopheline mosquitoes after seven years use in Tanzanian villages. Malaria J.

[13] Tungu PK, Malima R, Mosha FW, Lyimo I, Maxwell C, Kaur H (2015). Evaluation of ICON Maxx, a long-lasting treatment kit for mosquito nets experimental hut trials against anopheline mosquitoes in Tanzania. Malaria J.

[14] Smith A, Webley D (1968). A Verandah-Trap Hut for studying the house-frequenting habits of mosquitos and for assessing insecticides III. The effect of DDT on behaviour and mortality. Bull Entomol Res.

[15] Kirstein OD, Faiman R, Knigin A, Gueta H, Stone A, Warburg A (2018). Studies on the behaviour and control of phlebotomine sandflies using experimental houses. Med Vet Entomol.

[16] Cabrera OL (2013). Casa experimental para el estudio del efecto de medidas de control en el intradomicilio sobre Lutzomyia longiflocosa (MSc Thesis).

[17] Wharton RH (1951). The behaviour and mortality of Anopheles maculatus and Culex fatigans in experimental Huts treated with DDT and BHC. Bull Entomol Res.

[18] World Health Organization (1975). Manual on practical entomology in malaria. Part II: methods and techniques.

[19] Pardo RH, Santamaría E, Cabrera OL (2017). Entering and exiting behaviour of the phlebotomine sand fly Lutzomyia longiflocosa (Diptera : Psychodidae) in rural houses of the sub-Andean region of Colombia. Mem Inst Oswaldo Cruz.

[20] Young DG, Duncan MA (1994). Guide to the identification and sand flies in Mexico, the west Indies, Central and South America (Diptera: Psychodidae).

[21] Achee NL, Youngblood L, Bangs MJ, Lavery JV, James S (2015). Considerations for the use of human participants in vector biology research a tool for investigators and regulators. Vector-Borne Zoonotic Dis.

[22] Pardo RH (2006). The ecology and control of cutaneous leishmaniasis in the sub andean region of south-west Colombia (PhD Thesis).

[23] Elnaiem DA, Elnahas AM, Aboud MA (1999). Protective efficacy of lambdacyhalothrin impregnated bednets against Phlebotomus orientalis, the vector of visceral leishmaniasis in Sudan. Med Vet Entomol.

[24] Alexander B, Jaramillo C, Usma MC, Quesada BL, Cadena H, Roa W (1995). An attempt to control phebotomine sand flies (Diptera Psychodidae) by residual spraying with deltamethrin in a Colombian Village. Mem Inst Oswaldo Cruz.

[25] Santamaría E (2016). Efecto de toldillos tratados industrial o manualmente con insecticida de larga duración en el control vectorial de la leishmaniasis cutánea en la región subandina de Colombia (PhD Thesis).

[26] Winkler MS, Tchicaya E, Koudou BG, Donzé J, Nsanzabana C, Müller P (2012). Efficacy of ICON(r) Maxx in the laboratory and against insecticide-resistant Anopheles gambiae in central Côte d&apos;Ivoire. Malaria J.

[27] 27. Guillet P, N&apos;Guessan R, Darriet F, Traore-Lamizana M, Chandre F, Carnevale P. Combined pyrethroid and carbamate &raquo;,» &reg;,® &sect;,§ &shy;,­ &sup1;,¹ &sup2;,² &sup3;,³ &szlig;,ß &THORN;,Þ &thorn;,þ &times;,× &Uacute;,Ú &uacute;,ú &Ucirc;,Û &ucirc;,û &Ugrave;,Ù &ugrave;,ù &uml;,¨ &Uuml;,Ü &uuml;,ü &Yacute;,Ý &yacute;,ý &yen;,¥ &yuml;,ÿ &para;,¶ two-in-one &raquo;,» &reg;,® &sect;,§ &shy;,­ &sup1;,¹ &sup2;,² &sup3;,³ &szlig;,ß &THORN;,Þ &thorn;,þ &times;,× &Uacute;,Ú &uacute;,ú &Ucirc;,Û &ucirc;,û &Ugrave;,Ù &ugrave;,ù &uml;,¨ &Uuml;,Ü &uuml;,ü &Yacute;,Ý &yacute;,ý &yen;,¥ &yuml;,ÿ &para;,¶ treated mosquito nets: field efficacy against pyrethroid-resistant Anopheles gambiae and Culex quinquefasciatus. Med Vet Entomol. 2001; 15: 105-12. 10.1046/j.1365-2915.2001.00288.x 11297094

[28] Roberts D, Duarte-Alecrim W, Hshieh P, Grieco J, Bangs M, Andre R (2000). A probability model of vector behavior effects of DDT repellency, irritancy, and toxicity in Malaria Control. J Vector Ecol.

[29] Thanispong K, Achee NL, Bangs MJ, Grieco JP, Suwonkerd W, Prabaripai A (2009). Irritancy and repellency behavioral responses of three strains of Aedes aegypti exposed to DDT and a-cypermethrin. J Med Entomol.

[30] Courtenay O, Gillingwater K, Gomes PAF, Garcez LM, Davies CR (2007). Deltamethrin impregnated bednets reduce human landing rates of sandfly vector Lutzomyia longipalpis in Amazon households. Med Vet Entomol.

